# Percutaneous Injection of Strontium Containing Hydroxyapatite versus Polymethacrylate Plus Short-Segment Pedicle Screw Fixation for Traumatic A2- and A3/AO-Type Fractures in Adults

**DOI:** 10.1155/2018/6365472

**Published:** 2018-03-05

**Authors:** Panagiotis Korovessis, Eva Mpountogianni, Vasileios Syrimpeis, Andreas Baikousis, Vasileios Tsekouras

**Affiliations:** Orthopaedics Department, General Hospital of Patras, Tsertidou Str. 1, 26224 Rio, Greece

## Abstract

**Introduction:**

Polymethacrylate (PMMA) is commonly used in vertebroplasty and balloon kyphoplasty, but its use has been associated with complications. This study tests three hypotheses: (1) whether strontium hydroxyapatite (Sr-HA) is equivalent to PMMA for restoring thoracolumbar vertebral body fractures, (2) whether the incidence of PMMA leakage is similar to that of Sr-HA leakage, and (3) whether Sr-HAis is resorbed and substituted by new vertebral bone.

**Materials and Methods:**

Two age- and sex-matched groups received short percutaneous pedicle screw fixation plus PEEK implant (Kiva, VCF Treatment System, Benvenue Medical, Santa Clara, CA, USA) filled with either Sr-HA (Group A) or PMMA (Group B) after A2- and A3/AO-type thoracolumbar vertebral body fractures. The Visual Analog Scale (VAS) score and imaging parameters, which included segmental kyphosis angle (SKA), vertebral body height ratios (VBHr), spinal canal encroachment (SCE), bone cement leakage, and Sr-HA resorption, were compared between the two groups.

**Results:**

The average follow-up was 28 months. No differences in VAS scores between Groups A and B were observed at baseline. Baseline back pain in both groups improved significantly three months postoperatively. Anterior, middle, and posterior VBHr did not differ between the two groups at any time point. SKA was improved insignificantly in both groups. SCE decreased insignificantly in both groups on 12-month follow-up using computed tomography (CT). PMMA leakage was observed in one patient, while no Sr-HA paste leakages occurred. Sr-HA resorption and replacement with vertebral bone were observed, and no new fractures were observed.

**Conclusions:**

As all hypotheses were confirmed, the authors recommend the use of Sr-HA instead of PMMA in traumatic spine fractures, although more patients and longer follow-up will be needed to strengthen these results. This trial is registered with NCT03431519.

## 1. Introduction

Polymethacrylate (PMMA) is commonly used in vertebroplasty (VP) and balloon kyphoplasty (BK) for osteoporotic and fresh thoracolumbar fractures. VP and BK can be used either alone or in combination with pedicle screw constructs. However, PMMA has been reported to be associated with undesirable properties such as high setting temperature, leakage (7–10%), lung and distal emboli, lack of osseointegration, and significant stiffness mismatch with bone leading to subsequent adjacent fractures or even refracture of the augmented vertebra [1]. In consideration of these potential complications, biological and bioactive bone substitutes (calcium phosphate, Sr-HA, etc.) have been used in an attempt to reduce the undesirable events associated with PMMA, while enhancing the mechanical stability of osteoporotic vertebral compression fractures. Compared with PMMA, significantly lower leakage rates have been reported with biologic and bioactive bone cements [1–7].

Strontium (Sr) is an antiosteoporosis agent, which has dual effects on bone metabolism [8]. Sr restores the bone turnover balance, especially when the treatment of bone fractures caused by osteoporosis is challenging [9, 10].* In vitro* studies have shown that Sr acts through the calcium-sensing receptor to increase the mRNA level of osteoprotegerin and decrease the mRNA levels of the receptor activator of nuclear factor-kappaB ligand [11]. Furthermore, Sr promotes bone formation by stimulating the differentiation of osteoblasts, as well as by blocking bone resorption through inhibition of osteoclast differentiation [11]. A 10-year clinical trial reported that Sr reduces the risk of vertebral and nonvertebral fractures and increases bone mineral density [11].

Sr-HA exhibits radiopacity three times greater than that of cortical bone and thus has enhanced visibility compared to bone. This is an advantage from the perspective of clinical imaging when it is used to assess implant placement and osseointegration at the bone-implant interface [12, 13].

Previous investigations have not identified any adverse reactions associated with Sr-HA use (such as foreign body reaction, inflammation, or bone necrosis). This is likely because of the nontoxicity of bisphenol-A bis(2-hydroxypropyl)methacrylate (BISGMA) and the lower setting temperature of Sr-HA [6, 12, 14].

Clinical studies reporting the use of Sr-HA in VP and BK for vertebral compression fractures are lacking [5]. An experimental study showed that Sr-HA facilitated reconstruction and maintenance of vertebral body height. It was also reported that Sr-HA is incorporated in the fractured vertebral body during bone remodeling by 3 to 6 months after the VP [5]. In an animal study, new lamellar bone grew onto the Sr-HA, soon after surgery [15].

Since biologic and bioactive cements do not provide immediate stability like PMMA can, several authors are currently performing BK and VP procedures for A2- and A3/AO-type thoracolumbar fractures using titanium stents and PEEK devices, supplemented with short-segment pedicle screws, to increase immediate stability [16–18].

Taking into consideration the biological properties of Sr-HA bioactive bone cement, the authors of this study have been using Sr-HA with PEEK implants together with percutaneous pedicle screw fixation in selected young adult patients with fresh, severely compressed, A2- and A3/AO-type thoracolumbar fractures. The aim of this preliminary comparative study was to examine the short- to medium-term efficacy of percutaneous vertebral body reconstruction by vertebral body augmentation with Sr-HA paste plus short-segment pedicle screw fixation in fresh fractures, as well as evaluate Sr-HA resorption/substitution. The hypotheses tested in this prospective comparative controlled study were as follows: (1) whether Sr-HA is equivalent to PMMA for restoring the fractured thoracolumbar vertebral body, (2) whether leakage of Sr-HA is less than that of PMMA, and (3) whether Sr-HA is completely resorbed and replaced by cancellous bone.

## 2. Methods

### 2.1. Patients

This study was approved by the institutional ethical committee and all patients gave written informed consent. From April to December 2013, thirty-eight (38) consecutive adult female patients were selected and divided into two groups, each consisting of 19 age-matched individuals. Each of the subjects had a single, severely (>40%) compressed A2- and A3/AO-type thoracolumbar (T10-L3) fracture, without any serious concomitant injuries. For treatment of the fractures, the subjects received VP with PEEK and either Sr-HA (Group A) or PMMA (Group B). A total of 8 patients (4 in each group) were excluded from the final evaluation for the following reasons: one patient was excluded because her intraoperative biopsy revealed metastatic disease; six patients were excluded because they were not available for further evaluation after the 3-month follow-up; and one patient was excluded because of a deep-tissue infection. Finally, each of the two groups included 15 age-matched (*P* = 0.52) adult female patients. Group A included 15 women aged 45.7 ± 8 years (range: 38–53 years), and Group B included 15 women aged 46 ± 6 years (range: 40–52 years) at the time of the index surgery. The body mass index (BMI) of the subjects in Group Α averaged 28 ± 5 (range: 19–39) and in Group B averaged 26 ± 3 (range: 22–30) (*P* = 0.32). All patients from both groups were attended by the same senior spine surgeon and underwent percutaneous short pedicle screw fixation plus vertebroplasty with PEEK implants (Kiva, VCF Treatment System, Benvenue Medical, Santa Clara, CA, USA) filled with either Sr-HA paste (Neogel, Teknimed, Vic-en-Bigorre, France) (Group A) or low-viscosity PMMA (Group B).

Patients were excluded from the study in cases of polytrauma, neurologic impairment, spinal deformity, known malignancy, and previous fracture or surgery in the same or adjacent vertebrae. Back pain intensity was recorded using the VAS scoring system (10-point scale).

Patients were randomly assigned to receive either Sr-HA or PMMA, and the process was blinded by the following method: the second author randomized the patients to receive either PMMA or Sr-HA without knowledge of the patients' names. The surgeons were unaware of which patients would receive Sr-HA or PMMA.

The operation was performed in the prone position. Two multiaxial, cannulated pedicle screws with diameters of 6-7 mm were inserted into the vertebrae immediately superior and inferior to the fractured vertebra via stab incisions using an image intensifier. One PEEK implant was introduced unilaterally through the pedicle to reduce the likelihood of vertebral body collapse, and subsequently the filling material, PMMA or Sr-HA, was injected. A column of either PMMA or Sr-HA was constructed inside the PEEK implant. Next, two longitudinal, appropriately contoured rods were percutaneously inserted and assembled with the pedicle screws in each side. Supine, anteroposterior, and lateral digital roentgenograms of the thoracolumbar spine were taken on admission. Standing digital roentgenograms of the whole spine were taken on the second postoperative day, 6 months postoperatively, and at the final observation (average follow-up of 28 months). CT scans were performed preoperatively and at 12 months postoperatively to evaluate the spinal canal remodeling, the Sr-HA resorption, and the bone healing state in the fractured vertebral body.

The following roentgenographic parameters were measured at the time of admission and postoperatively: (a) segmental kyphotic deformity (SKD) (defined by the angle formed from the lines drawn on the lower endplate of the intact vertebra inferior to the fractured vertebra and the upper endplate of the adjacent vertebra superior to the fractured vertebra), (b) anterior vertebral body height ratio (AVBHr), (c) middle vertebral body height ratio (MVBHr), (d) posterior vertebral body height ratio (PVBHr) (vertebral body height ratios are equal to the fractured vertebral body height divided by the average of the vertebral body heights of the adjacent intact vertebrae superior and inferior to the fractured vertebra), (e) refractures, and (f) adjacent or remote vertebral fractures.

On axial CT scans, the percentages (%) of spinal canal encroachment (SCE) and spinal canal clearance (SCC) (the narrowest anteroposterior spinal canal diameter divided by the average anteroposterior diameter of the two adjacent noninjured vertebrae) were evaluated.

**Figure 1:**
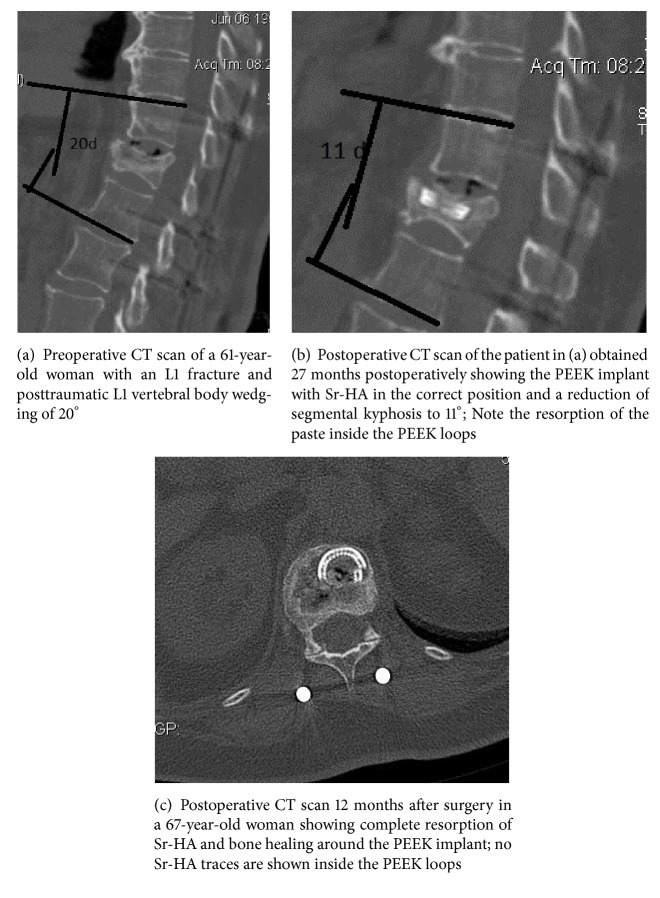


One senior orthopedic spine surgeon and one senior orthopedic radiologist were asked to evaluate the following parameters on axial CT scan slices: cement (Sr-HA or PMMA) leakage and Sr-HA resorption and bone substitution within the cylinder formed by the implanted PEEK loops. Since there is no available method for quantitative or qualitative evaluation of bone cement resorption, the evaluation of Sr-HA resorption and cancellous bone formation (Group A) inside the column constructed by the PEEK loops was rated as present (+) or absent (−) based on different axial CT scan slices taken 12 months postoperatively (Figures 1(a)–1(c)). Patients were encouraged to walk wearing a 3-point fixation brace from the first day following surgery and for a period of 6 weeks.

### 2.2. Statistical Analysis

Statistical analysis was performed with paired (change of variables in the same group) and unpaired (change of a variable in different groups) *t*-tests for changes in every radiographic parameter. The kappa value for agreement was used for the radiological evaluation of Sr-HA resorption only, since the digital roentgenograms and CT scans are highly reliable. The interobserver reliability was measured by the kappa values. Kappa values between 0.61 and 0.80 were considered to indicate “substantial agreement.”

### 2.3. Implant Characteristics

Sr-HA paste is a radiopaque, osteoconductive, and osteocompatible bone substitute that was used in this study, together with the PEEK implant, for vertebral body augmentation in all 15 patients of Group A. Sr-HA consists of highly pure synthetic, fully resorbable, nanocrystalline hydroxyapatite (HA) with strontium (Sr) and water. Sr-HA is gradually resorbed and replaced by bone during the remodeling process, and the strontium-developed drug acts as an effective antiosteoporotic therapy in postmenopausal women with osteoporosis [19, 20].

Previous studies have reported the occurrence of osteogenesis early after the implantation, while the histological integration of Sr-HA in sheep's vertebrae revealed excellent osteogenic and osteoconductive properties. No adverse events (cytotoxicity or hemolysis), rejections, or osteolyses have been recorded [6, 21, 22]. The reported compressive strength of Sr-HA is 40.9 MPa, which is 2 to 9.9 times lower than that of PMMAs (80–396 MPa) and 2–8 times higher than that of cancellous bone (6–24 MPa) [14]. Its bending strength is 31.3 MPa, which is 50% lower than that of PMMAs (67–72 MPa) and 10 times stronger than that of cancellous bone (3 MPa) [12, 14].

## 3. Results

All patients in both groups were followed up for an average of 28 months (range: 24–33 months).

The* operative time* in both groups averaged 65 minutes (range: 55–80 minutes). The* fluoroscopy exposure time* ranged from 1.05 to 4.2 minutes, with an average time of 1.3 ± 1.14 min, and this was similar in both groups (*P* = 0.72). The* volume of injected liquid *of Sr-HA (Group A) per vertebra averaged 1.5 ml (range: 1-2 ml) and did not differ from PMMA with average volumes of 2 ml (range: 1–2.5 ml) (Group B) (*P* = 0.64).

No significant amount of perioperative* blood loss *was observed in either group. No significant decrease in hemoglobin was observed postoperatively, and no blood transfusion was given to any patient in both groups. The* hospital stay *in both groups averaged 2 days (range: 1–3 days).

Baseline* VAS back pain *scores in both groups improved significantly by 3 months postoperatively (*P* < 0.000). Improvements of VAS scores in Group A averaged 2 ± 3, and the average in Group B was 1.6 ± 2. There was no difference in VAS changes between the two groups (*P* = 0.7). No further significant changes in VAS score were seen in the patients of either group.

**Figure 2:**
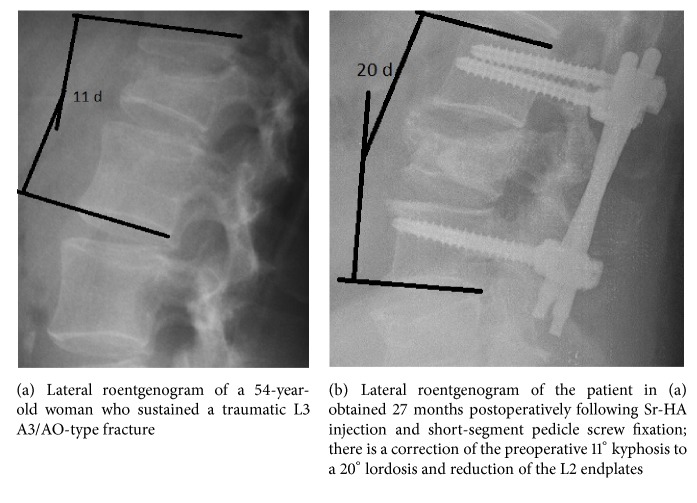


The* kappa values* among the two observers after evaluating the roentgenographic and CT parameters ranged from 0.96 to 0.98. The baseline and follow-up radiographic and CT scan parameters values did not differ significantly between the two groups (Table 1, Figures 2(a) and 2(b)).

PMMA leakage without sequelae was observed in one patient in Group B, while no Sr-HA paste leakage was observed in Group A. No loss of correction of SKA, AVBHr, and PVBHr and no change of SCC were measured at the latest observation point (average follow-up of 28 months) in both groups (Table 1). Sr-HA resorption and replacement with vertebral bone were observed by 12 months postoperatively in all the spines of Group A (Figures 1(a), 1(b), and 1(c)). No adjacent or remote new fractures were observed in the latest follow-up (average follow-up of 28 months).

## 4. Discussion

This preliminary comparative study examined the short- and medium-term efficacy of percutaneous VP with PEEK and Sr-HA or PMMA plus short-segment pedicle screw fixation for single fresh A2- and A3/AO-type thoracolumbar fractures. We also evaluated the amount of Sr-HA resorption and substitution with cancellous bone. All three hypotheses in this study were confirmed: (1) Sr-HA and PMMA equally restored the fractured thoracolumbar vertebral body angulation and heights, (2) bone cement leakage in VP with Sr-HA was less than that with PMMA, and (3) Sr-HA paste was resorbed and replaced by cancellous bone.

Previous clinical studies showed that percutaneous VP with PEEK and low-viscosity PMMA for osteoporotic vertebral body fractures in elderly patients exhibited PMMA containment [13, 16]. However, because of the exothermic reaction and heat released during the PMMA hardening process, an active membrane is created around the PMMA cement, which is considered to be a potential disadvantage of PMMA osseointegration [3, 23, 24]. It seems that osseointegration provides a critical advantage of Sr-HA over PMMA. This advantage of Sr-HA may result in better long-term outcomes and fewer potential adverse reactions than those reported with PMMA, in cemented hip and knee arthroplasties. Previous studies reported no adverse reactions associated with Sr-HA use, probably because of the nontoxic nature of bisphenol-A bis(2-hydroxypropyl)methacrylate (BISGMA) and the lower setting temperature of Sr-HA [6, 12, 14].

HA is a biocompatible and osteoconductive material. The mechanism of formation and strengthening of the bone-HA interface has been studied using high-resolution transmission electron microscopy and energy-dispersive X-ray analysis [25]. It was suggested that the transition of crystalline to amorphous HA is the first critical step in bone-to-implant bonding. The osseointegration of an implant as a function of the biological response to HA can be significantly enhanced by pharmaceutical agents [26, 27]. It was found that apatites obtained by partial substitution of Ca^++^ by Sr yield higher solubility compared to pure HA [28]. Both Sr and Ca^++^ have common chemical properties, while Sr is characterized as “a bone seeking element” having a similar charge/size ratio to Ca^++^.

A previous study showed an increase in bone mass following oral intake of Sr-HA for osteoporosis [9]. Sr is beneficial for bone health in postmenopausal osteoporotic women, because it assists in the replication of preosteoblastic cells promoting bone formation [29] and, to a lesser extent, decreases bone resorption* in vivo *[30].

Oral administration of Sr salts results in adsorption of Sr ions on the apatite surface or in the substitution of Ca^++^ ions in the apatite crystal lattice at low ion exchange rates. Only about 10% of the Ca^++^ ions are substituted by Sr ions [31]. The procedure of ion exchange is more profoundly realized in new bone during remodeling. There is some controversy regarding the beneficial effects of Sr on bone because it is dose-dependent, and although low doses stimulate bone formation, high doses have deleterious effects on bone mineralization [9, 32–34]. Increased attention has been paid to the repair of osteoporotic fractures by Sr-modified bioceramics with improved osseointegration capabilities. Moreover, biocompatible Sr-doped HAs enhance the peri-implant bone formation more efficiently than the administered strontium [35].

Recently, Li et al. [36] published a thought-provoking study of doped hydroxyapatite (Sr-HA) in ovariectomized rats, comparing HA and Sr-HA. In this study, Li et al. [36] clearly showed that Sr-HA is a promising material for bone tissue engineering, because it promotes osteogenesis and improves the trabecular microarchitecture under osteoporotic conditions. The osteoconductive properties of different Sr-doped biocomposites have been investigated in numerous bone defects [37, 38]. It has been reported that the implantation of Sr-containing HA materials promotes bone repair and healing in both normal [39] and ovariectomized [36, 40] animals.

A previous study regarding the use of VP for osteoporotic single spine fractures revealed that Sr-HA is incorporated during the bone remodeling as early as 3 to 6 months after implantation [5]. In an animal research study, newly formed bone grew onto the Sr-HA by 4 months following surgery [15]. Complete Sr-HA resorption was documented on CT scans 12 months postoperatively in all 15 patients of Group A, justifying the previously mentioned study. This means that the Sr-HA ingredients (hydroxyapatite and strontium) are resorbed without interference during the vertebral bone healing process and probably induce osteogenesis.

Another advantage of the use of bioactive cements is their lower leakage rate [5, 13]. A leakage rate of 22% was reported with the use of PMMA in fresh thoracolumbar vertebral body fractures, whereas the leakage rate with calcium phosphate was 15% [13]. A clinical study reporting on the use of Sr-HA in VP for the augmentation of single osteoporotic thoracolumbar fractures revealed maintenance of the vertebral body height with only 3 (13%) cases of slight Sr-HA leakage into the spinal canal, but none of the patients developed any neurologic sequela [5]. In the present study, the leakage rate with PEEK was 7.5% when using PMMA and 0% when using Sr-HA.

The addition of pedicle screws together with the PEEK implant secured the vertebral body stiffness against compression and rotational forces acting across the thoracolumbar spine immediately following surgery and thus, at least theoretically, enhanced the process of bone healing and new bone formation. There were no reactions, either local or systemic, to Sr-HA injections in any of the patients.

In contrast to Sr-HA, which is a resorbable bioactive bone cement, PMMA is not resorbable. Taking into consideration the extensive experience gained in clinical practice with PMMA and its low cost, efforts have been made recently to improve the bioactivity of PMMA [41]. Researchers have attempted to improve PMMA bioactivity and osseointegration by incorporating Sr-containing borate bioactive glass (SrBG) as a reinforcement phase and bioactive filler for the PMMA cement [41]. The prepared SrBG/PMMA composite cements showed evidence of significantly decreased polymerization temperatures, when compared with PMMA alone. The composite also retained the properties of appropriate setting times and great mechanical strength. The bioactivity of the SrBG/PMMA composite cements was confirmed* in vitro*, as evidenced by ion release (Ca^+2^ P, B, and Sr) from SrBG particles. It has been demonstrated* in vitro *that SrBG incorporation may promote adhesion, migration, proliferation, and collagen secretion by cells. Consequently, the SrBG/PMMA composite cement may be a better alternative to cement made from PMMA alone, in clinical applications, and it has promising applications in minimally invasive orthopedic surgery.

A limitation of this study is that it is a pilot study, with a small (*n* = 30) number of patients. Further, it has limited power, since the results may be subject to a Type II error.

## 5. Conclusions

Based on the results of the present preliminary study and taking into consideration the small number of individuals included, the authors recommend using Sr-HA bioactive bone cement instead of PMMA, supplemented by short pedicle screw construction, in adults with osteoporotic and fresh traumatic thoracolumbar fractures. However, further studies, with a greater number of patients and longer follow-up, are needed.

## Figures and Tables

**Table 1:** Comparative presentation of radiological changes observed in Group A and Group B preoperatively till the last follow-up.

Parameters	PREOP		GrA/GrB	Postop		GrA/GrB	3 months		GrA/GrB	6 months		GrA/GrB	Last follow-up		GrA/GrB
	GrA	GrB	*P* value	GrA	GrB	*P* value	GrA	GrB	*P* value	GrA	GrB	*P* value	GrA	GrB	*P* value
AVBHr	0.59 ± 0.11	0.63 ± 0.14	0.48	0.79 ± 0.12	0.87 ± 0.15	0.22	0.75 ± 0.12	0.86 ± 0.17	0.1	0.7 ± 0.15	0.9 ± 0.20	0.089	0.71 ± 0.16	0.91 ± 0.19	0.09
MVBHr	0.53 ± 0.12	0.68 ± 0.16	**0.054**	0.66 ± 0.08	0.86 ± 0.13	**0.06**	0.73 ± 0.15	0.8 ± 0.20	0.24	0.63 ± 0.2	0.8 ± 0.12	0.21	0.6 ± 0.18	0.82 ± 0.13	0.23
PVBHr	0.95 ± 0.15	0.97 ± 0.18	0.77	0.97 ± 0.07	1 ± 0.09	0.069	1 ± 0.12	1 ± 0.11	0.13	0.98 ± 0.13	1 ± 0.18	0.4	1 ± 12	1.1 ± 0.17	0.45
^+^SKA	14.7 ± 8.7	13.9 ± 8	0.81	8.6 ± 7	9 ± 4	0.88	13 ± 8	10 ± 6	0.40	10 ± 6	12 ± 8	0.65	12 ± 5	14 ± 9.8	0.66
SCE (%)	22 ± 17	26 ± 19	0.46							15 ± 13^*∗*^	10 ± 15^*∗*^	0.49			

^*∗*^SCE values on CT scan 12 months following surgery. GrA: Group A; GrB: Group B; AVBHr: anterior vertebral body height ratio; MVBHr: middle vertebral body height ratio; PVBHr: posterior vertebral body height ratio; SCE: spinal canal encroachment. ^+^SKA in Cobb degrees.
